# Poverty alleviation policies, programs and practices for people with disabilities: A scoping review and recommendations

**DOI:** 10.1371/journal.pone.0323540

**Published:** 2025-05-13

**Authors:** Sally Lindsay, Janice Phonepraseuth, Sarah Leo

**Affiliations:** 1 Bloorview Research Institute, Holland Bloorview Kids Rehabilitation Hospital, Toronto, Ontario, Canada; 2 Department of Occupational Science and Occupational Therapy, University of Toronto, Toronto, Ontario, Canada; 3 Rehabilitation Sciences Institute, University of Toronto, Toronto, Canada; Hong Kong Shue Yan University, HONG KONG

## Abstract

**Background:**

People with disabilities have a higher prevalence of living in poverty compared to people without disabilities, which largely results from the challenges, barriers, inequalities and discrimination they often encounter. However, little is known about relevant policies, practices, and anti-poverty interventions that could facilitate a better quality of life for people with disabilities.

**Methods:**

A scoping review following the Joanna Briggs Institute methodology was used to explore the existing practices, policies and interventions to address poverty among people with disabilities. The search involved six international databases: Ovid Medline, Healthstar, PsychINFO, Econlit, Scopus and Web of Science where two reviewers screened 4548 studies for inclusion.

**Results:**

Thirty-seven studies were included in the review, which spanned across 20 countries. Our review noted the following key trends: (1) poverty alleviation policies; (2) programs and practices to address poverty (e.g., benefits, barriers and factors affecting access); and (3) cash transfers, especially their impact and factors affecting transfers.

**Conclusions:**

The findings of this review underscore the potential value of poverty alleviation strategies and policies for assisting people with disabilities. The results could help to inform guidelines and recommendations for policies, practices, and interventions to help alleviate poverty among people with disabilities.

## Introduction

Socio-economic status, including income level and poverty, is an important aspect of the social determinants of health [1,2]. Research consistently shows that people with a steady income and higher socio-economic status are less likely to live in poverty and often have better health outcomes compared to those living in poverty [3]. Experiencing poverty can affect health, quality of life and access to resources [4]. Thus, understanding how to address the socioeconomic inequities impacting health is critically important. Ending poverty in all its forms, which includes inequity and other deprivations (e.g., social, health, and financial), is a primary objective of the United Nations Sustainable Development Goals [5].

Research consistently shows a link between poverty and disability [6,7]. People with disabilities, who comprise approximately 15% of the global population, are significantly more likely to live in poverty compared to people without disabilities [8–10]. For example, a study focusing on data from 15 countries found that people with disabilities and their households were significantly worse off in multiple dimensions of poverty and experienced more deprivations than people without disabilities [11]. People with disabilities often encounter additional costs for daily living, health care, transport, assistive devices and personal assistance [12,13]. Having a disability can exacerbate poverty due to barriers, discrimination, and inequalities [14,15]. For example, individuals with disabilities have persistently higher unemployment rates compared to people without disabilities [16–18]. It is evident that people with disabilities have a strong need for poverty alleviation strategies due to their susceptibility to poverty. Additionally, living in poverty can often create or worsen disabilities due to riskier and hazardous living or working conditions (e.g., malnutrition, exposure to disease, inadequate access to treatment, and lack of support), and barriers in accessing health care services [7,8,13,19]. Given the high percentage of people with disabilities living in poverty, it is essential to understand effective strategies to address poverty alleviation to enhance their social, economic and physical well-being.

### Benefits of poverty alleviation interventions

There are many potential benefits of poverty alleviation and social protection policies and programs [20]. Research consistently shows a strong negative correlation between poverty and social expenditures [13,21], which demonstrates the importance of implementing such measures to address poverty. Social programs and income transfers are an important strategy aiming to help protect human rights [22], ensuring people are financially secure, have enough food to eat and pay for essential expenses.

A common strategy for poverty alleviation is social protection and income/cash transfers [23,24], which are essential for disability inclusion [25]. Social protection includes initiatives to address poverty, improve living conditions and alleviate risk [26]. Social protection programs can have a direct impact on the lives of people with disabilities through reducing poverty and enabling participation in economic activities, reducing vulnerability through asset accumulation and a more stable cash flow [10]. The United Nations Convention on the Rights of Persons with Disabilities emphasizes the right to inclusion in social protection programs [27], emphasizing how essential social protection programs are in alleviating poverty, particularly for people with disabilities. Cash transfers can occur in the form of disability grants, social pensions, child support grants and transfers to poor households, among others [22]. They can help to improve household resources, alleviate poverty and reduce household financial burden [22,25]. Social programs and income transfers are an important strategy aiming to help protect human rights [5,10], and ensuring financial and food security. Indirect benefits of poverty alleviation policies and programs include better access to health care, education, social supports, food security, nutritional status and increased self-esteem and dignity, among others [10].

### Challenges with accessing poverty alleviation strategies

Although there are many potential benefits of poverty alleviation strategies, several challenges also exist with accessing such supports for people with disabilities [15]. Some researchers argue that programs and policies aiming to reduce poverty, especially for those with disabilities, often fail to reach those most in need [8]. Indeed, people with disabilities often experience exclusion from livelihood programs that are not properly adapted to their particular needs [28]. For example, some research shows that people with disabilities encounter challenges with accessing social programs, often because they do not exist, they are unaware of them; they are poorly implemented, or are insufficiently funded [8,29]. Thus, there is an urgent need to develop a better understanding of effective poverty alleviation policies, programs, and practices for people with disabilities to enhance their social inclusion and wellbeing.

### Novelty of this review

This review is relevant and novel in that it aligns with the United Nations Sustainable Development Goals aiming to end poverty and inequity [5]. Previous reviews on this topic have explored multidimensional poverty and disability [7], the extra costs of living with a disability [30], social protection programs in low and middle income countries [8], welfare-to-work programs for people with disabilities [31], the impact of disability benefits on employment [32,33], a mapping of financial support programs for children with neurodisabilities in Canada [28], income supports for parents of children with disabilities [34,35], and the need for a better understanding of policies and practices to address disability and poverty [9]. Many of these reviews focus on identifying and describing the availability of supports and not necessarily their impact. Moreover, a recent review on the experiences of poverty among people with disabilities [36] highlighted the urgent need for a better understanding of relevant practices and strategies for reducing poverty among people with disabilities. To our knowledge no reviews currently exist that explore the impacts of poverty alleviation policies, practices, and interventions for people with disabilities.

## Materials and methods

The research question for this review was: what practices, policies, and interventions exist to address poverty among people with disabilities? This scoping review followed the most recent best practices in scoping review methodology [37,38]. The key steps involved with the method include: preparation, organizing, and presenting the findings [38]. We also applied the guidelines of the Preferred Reporting Items for Systematic Reviews and Meta Analysis, extension for scoping reviews (PRISMA-ScR) checklist (see S2 File) [39]. A scoping review was applicable for this topic because this type of methodology intends to chart the size and scope of the literature while also noting relevant future directions [37].

### Search strategy

The search strategy was developed with the involvement of a research librarian and researchers with experience in disability, socio-economic status and poverty. The following six databases were searched: Ovid Medline, Healthstar, PsychINFO, Econlit, Scopus and Web of Science. The search involved the following key concepts: *poverty alleviation* (i.e., poverty reduction, poverty alleviation, social security, income transfer, supplemental income, cash transfer, social grant, income assistance, poverty intervention, social assistance, and social protection) and *disability* (i.e., disability, disabled persons, functional limitation, physical impairment, mobility impairment, sensory impairment, motor impairment, vision impairment, hearing impairment, and wheelchair user) (see S1 File for full search strategy). We also reviewed the references of all articles meeting the inclusion criteria for additional relevant articles.

Articles meeting the following criteria were included in the review: (1) a sample of people with disabilities (based on the definition from the International Classification of Functioning, Disability and Health: “an umbrella term for impairments, activity limitations and participation restrictions”) [40]; (2) empirical research (quantitative or qualitative methodology) involving at least one finding focusing on policies, practices, interventions or solutions to reduce poverty (incorporating both absolute and relative definitions; involving at least one monetary related finding) for people with disabilities; and (3) published up to June 2024 in a peer reviewed journal, without language restrictions. We excluded grey literature and non-peer reviewed articles as they can include bias. Focusing on empirical, peer-reviewed literature is critical for evidence informed decision making and policy development [41,42]. We excluded articles on school-to-work interventions because reviews covering that topic already exist [43].

**Fig 1:**
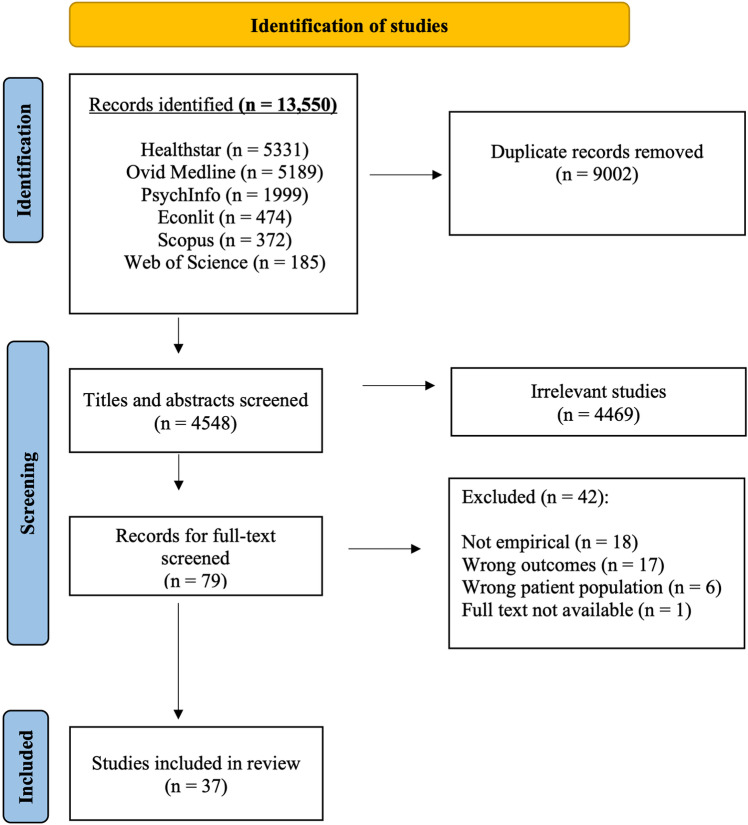
Overview of the search process.

The first author, experienced in review methodology, conducted the search and imported the articles into *Covidence* [44], a software supporting reviews including screening and data extraction. After we removed the duplicates, two researchers screened 4548 titles and abstracts where 4469 were irrelevant and excluded (see Fig 1). Three researchers independently reviewed the 79 full text articles, where 37 met the inclusion criteria for our review. Any articles where it was unclear whether they met the inclusion criteria were re-examined and discussed until consensus was reached amongst the research team.

### Data extraction and synthesis

For the data extraction phase, we first developed and piloted a data extraction form. Pairs of authors independently extracted the data for each article, then compared and discussed until consensus was reached. Data included relevant information about each study (including authorship, year of publication, country, recruitment setting, and design), participants (sample size, age, income, disability type, and social demographics) and outcomes (policies, practices and poverty alleviation interventions). Once the data extraction table was complete, the research team discussed the key findings across the studies. To organize the findings, we applied an inductive open coding approach [38], which involved reviewing the findings within and between each study while making notes of the common patterns and trends of poverty alleviation strategies for people with disabilities. In the final stage of the scoping review, the presentation and synthesis phase, we developed a narrative overview of the results while also following the reporting guidelines of the PRISMA-ScR checklist (see S2 File) [39].

## Results

### Study characteristics

Thirty-seven studies met the inclusion criteria for our review. The studies spanned across 20 countries (i.e., Belgium [45,46], Brazil [47,48], China [49,50], Ecuador [51], Ghana [52–54], Guatemala [55], Kenya [22], Korea [56], Italy [57], the Maldives [24,58], Malawi [25,59], Mexico [55], Nicaragua [55], Nigeria [60], Norway [61], South Africa [62–67], Tanzania [68], Uganda [69], the United States [70–75]) and Zambia [25]) over a 16-year period (see Table 1). Three studies focused on several countries within their analysis [23,76,77]. Twenty-three studies used a quantitative design (i.e., randomized experiment [61], quasi experiment [58,75], case control study [67], secondary analysis of datasets [23,25,45,46,48,49,56,63,65,70,73,74,76,77], questionnaire [50,72], cross sectional retrospective study [47], simulation modeling [57], and case study [51]). Nine studies used a qualitative design (i.e., interviews [53,54,59,60,62,66,68,69], and focus groups [52]). Five studies had mixed methods [22,24,55,64,71]. Types of participants involved people with disabilities, households with a member with a disability, key informants, social entrepreneurs, bank managers and policy/grant implementation decision-makers.

**Table 1:** Overview of promising practices, policies and interventions to address poverty among people with disabilities.

Author (country of research focus)	Objective	Sample characteristics	Design and analysis (main outcome)	Key findings^*^	Recommendations
Abdille and Mbataru 2019 (Kenya) [22]	To assess the impact of cash transfers on the economic well-being of people with severe disabilities in Kenya	183 households with a member with a severe disability 19 senior officials working in sub-counties’ social development offices -no sample characteristics reported	Mixed methods: surveys and interviews (Cash transfer, economic wellbeing)	-Cash transfers supported household income, health care access, investment in productive activities and empowerment which had significant effect on the economic wellbeing of people with disabilities -79.4% of the changes in economic wellbeing of persons with disabilities were due to cash transfer and its impacts on household income, healthcare access investment in productive activities, empowerment of the beneficiary	-Government should increase monthly disbursements given to people with disabilities -Cash transfer programs should have follow-ups so people with disabilities can be supported to expand their income generation activities -Cash transfer programs should partner with other development programs to extend the financial and nonfinancial supports to people with disabilities
Agovino and Ferrara 2017 (Italy) [57]	To assess whether civilian disability pensions can address poverty	1,640,000 non-employed disabled people -avg. household income $35,018 (disabled); $41,104 (non-disabled) -disability and gender not reported	Simulation modeling (dynamic stochastic general equilibrium model) of business cycle (household income, poverty line)	-In the long-term a minimum increase of civil disability pensions allows households with a disabled member to consume more and exit from poverty -Helps reduce income inequality	-More alternative strategies to address poverty for people with disabilities
Arkorful et al. 2020 (Ghana) [53]	To assess perspectives of disability fund	130 people with disabilities, aged 0–60 + years (mean age: 48; 71.5% male) -10 key informants, aged 33–60; mean age: 46.5; 70% female) -income not reported	Interviews (perspectives of disability fund)	-Challenges with the disability fund include limitation information about the fund, access to the fund and disbursement delays, inadequate funds -Some people with disabilities found the fund helpful for meeting financial needs	-Financial literacy and management training for people with disabilities -Separate fund management from political structures
Banks et al. 2024 (Maldives) [58]	To explore the impact of disability allowance on financial wellbeing	616 respondents -141 intervention (mean age 37.5; 34.8% female; 20.3% in poverty) -269 control group (without disabilities) (mean age 44.6; 58.4% female, 11.7% in poverty) -Control group 2 (206 with disabilities, not enrolled in benefit: mean age 48.7; 64% female, 16.1% in poverty)	Quasi experimental design (per capita expenditure per day, poverty headcount, poverty gap)	-Did not have an observable impact on reducing poverty gap -Disability allowance had a decreased likelihood of using harmful coping mechanisms in response to food insecurity -Disability allowance was attributed to an increase in proportional consumption of food compared to control group 2	-Additional programs may be needed to address inequalities experienced by people with disabilities -Greater amount for disability allowance
Beisland and Mersland 2017 (Ecuador) [51]	To compare characteristics of disabled versus non-disabled clients in a microbank	281 respondents, mean age: 43.3; -96 clients with disabilities, mean age: 46.5; 35% female; average monthly income: $511 -average net assets: $11,937 -185 without disabilities -mean age: 41.59; 60% female; avg. monthly income: $457 -average net assets: $11,614	Case study and secondary analysis of survey data and bank records (income, net worth, creditworthiness)	-Disabled clients are more often male, less likely to be living with a partner, have fewer children and are older compared to those without disabilities -Persons with disabilities do not access microfinance institutions due to low income and are perceived to be risky clients	-It is important to adapt microloans to the needs of people with disabilities -Persons with disabilities should be empowered and included in mainstream microfinance institutions -Equity lens and equality lens needed for more positive and optimistic view of persons with disabilities
Berry and Caplan 2010 (US) [70]	To examine the factors that influence employment and earnings growth for supplemental security income beneficiaries who participated in the vocational rehabilitation program	3046 participants with disabilities (59.8% male, 40.2% female; 78.6% white, 18.8% African American, 1.6% Asian/Pacific Islander), ages 16–25 years -mean age and income not reported	Secondary analysis of administrative databases (poverty, unemployment, education)	-For social security income participants with sustained employment African American vocational rehabilitation consumers had higher earnings when compared to other race groups -College or university training was positively associated with earnings growth over time -Post-secondary training and job placement services decreased the odds of employment by 29% and 15%, respectively -Supported employment demonstrated increased odds of employment by 1.14	-Develop more insight into postsecondary education experiences of vocational rehabilitation consumers -Job placements may require additional supports to be effective -Consider offering individualized attention to vocational rehabilitation consumers in supported employment programs to ensure sustainable employment outcomes
Caminada et al. 2021 (Various Countries) [23]	To analyze the effectiveness of social transfers and income taxes in alleviating poverty	49 countries -no other sample characteristics reported	Secondary analysis of Luxembourg income study (income poverty)	-15% of the population is helped of poverty via tax/benefits -Disability/survivor scheme accounts for 81% of poverty reduction -Social transfers reduce poverty by 17.7% -Income and payroll taxes increase poverty by 2.3%	n/a
Campos et al. 2022 (Brazil) [47]	To examine the continuous cash benefit program for children and youth with disabilities	332 children with mental and behavioural, nervous system and congenital disabilities under 16 years -62.65% male; mean age: 7.32 -income not reported	Cross sectional, retrospective study (prevalence of disease)	-Predominance of male, preschoolers and illiterate individuals in the benefit program -Program facilitates social inclusion	-Update continuous cash benefit legislation
Connor and Bent-Goodley 2016 (Tanzania) [68]	To understand how social entrepreneurs can act as a poverty alleviation strategy in Zanzibar Tanzania	15 women social entrepreneurs (aged 19–54; mean age 38) -disability type not reported	Interviews (poverty alleviation strategies; life experiences as social entrepreneur)	-Women social entrepreneurs were successful in building educational opportunities for girls and low-income women, evolving in business to meet change and economic growth and empowering vulnerable populations and people with disabilities	-Engagement with local communities to address and promote economic stability
Glendening et al. 2018 (US) [71]	To examine relationships between disabilities and social security when families enter emergency shelters and how housing interventions affect social security	1857 families (mean age: 29; 92% female, median annual income $7440) -34.1% of families reported a disability	Longitudinal study (long-term housing subsidies, short-term rapid re-housing subsidies, and project-based transitional housing) Interviews (disability status, housing stability, social security, self-sufficiency)	-Among families reporting disabilities, social security receipt predicted fewer returns to emergency shelter and more income, despite less work -Offers of long-term housing subsidies increased receipt of social security	-Long term housing subsidies as a tool for increasing access to disability income
Hameed et al. 2023 (Maldives) [24]	To explore coverage of disability allowance in Maldives and factors affecting update	402 people with disabilities (mean age: 43.1; 44% male -income not reported	Mixed methods: secondary analysis of population-based survey (coverage of disability allowance) and interviews (experiences of accessing the allowance)	-25.6% of people with disabilities in Maldives are receiving the disability allowance -Coverage was lowest for women, older adults and people living in the capital city, wealthier households and people with sensory impairments -Factors affecting uptake of allowance included lack of information, perceptions of disability and eligibility criteria, geographical and financial factors	-Raising awareness about the existence of social protection programs and how to apply is important but may be insufficient for improving coverage without additional efforts to widen the knowledge of eligibility criteria and addressing stigma of receiving such benefits
Jiya et al. 2022 (Malawi) [59]	To explore preparedness and accessibility of financial services for people with disabilities	10 managers of commercial banks aged 26–45 years (80% male; mean age: 39 years old -income and disability type not reported	Interviews (perspectives on extending financial services for people with disabilities)	-Themes included: inclusion policies and accessibility; staff training for diverse customers; financial literacy events -barriers to financial inclusion still exist (business model; inaccessible technology; communication barriers)	-Banks and governments should partner to extend financial literacy to persons with disabilities and enhance financial services -Accessible communication and technology should be implemented into national policies
Karimu et al. 2024 (Ghana) [52]	To explore what is known about the District Assemblies Common Fund program and the benefits on beneficiaries’ livelihoods	35 people with disabilities, and 6 key informants, 54.3% male, -mean age and income not reported	Focus groups and interviews (knowledge of benefit program and its potential benefits)	-Program had positive outcomes on livelihoods of people with disabilities -People with disabilities were aware of the program -Barriers included: quality of purchased items, procurement issues; disparity in allocations and lack of transportation support	-Need for greater funds dedicated to disability fund -Needs assessment workers should communicate with welfare directors -greater accessibility and need for sign language interpreters
Kelly 2019 (South Africa) [62]	To explore the influence of disability-related grants on family practices, care and household composition	Study 1: 32 people with disabilities (75% women) -Study 2: 24 doctors; 12 social workers, nurses, and occupational therapists -mean age, income not reported	Ethnographic study (community based; and interviews) (family practices, care arrangements, and household composition)	-Disability grant income is shared within households -Contribution of a stable income provides opportunities for people with disabilities to have agency and secure care and support -Barriers to receiving the grant (family conflict); household pressure to retain grant and potential for neglect and abuse	-More community support for individuals with disabilities -Greater retraining and employment programs can help individuals with disabilities to find employment
Kostol and Mogstad 2014 (Norway) [61]	To understand how financial incentives induce disability insurance recipients to return to work	-no sample characteristics reported	Randomized experiment (insurance, capacity to work)	-Many disability insurance recipients have capacity to work can be induced by providing financial work incentives -Providing work incentives to disability insurance recipients may increase their disposable income and reduce program costs -Return to work program increased employment of disability insurance recipients by 8.5% and led to greater earnings	-Targeted policies may be most effective in encouraging disability insurance recipients to return to work
Lamont et al. 2023 (Brazil) [48]	To understand how the 2016 inclusion of people with disabilities act in Brazil act affected self-employment income and poor with disabilities	2555 people with disabilities ages 16–60 (mean age 43.2; 56% male -disability type not reported	Secondary analysis of Brazil’s Bolsa Familia (income, self-employment)	-Income of the employed improved after the act with the income gap between the employed and self-employed with disabilities growing wider after the law was passed -The law mostly benefited the poor with disabilities who were able to find employment	-Addressing stigmatization of disabled people could help the effectiveness of self-employment for people with disabilities -Increasing accessibility to transportation and communication
Lee and Choi 2018 (Korea) [56]	To examine antipoverty effects of income transfers in people with disabilities	6723 households with individuals aged ≥15 years -1211 with a disability (27% female; mean age 63.8; mean income $29,693) -5512 without a disability (72% male; mean age 55.8, mean $52,686)	Secondary analysis of national dataset (household income, poverty rate, income transfer)	-In households with a person with a disability, total income transfers decreased by 55.9% and 84.8% of the pre-transfer poverty rate and poverty gap. -Income transfers were more effective in reducing poverty levels than social insurance or private income transfers -Provision of means-tested programs was also more likely to decrease the likelihood of experiencing poverty than social insurance and private income transfers	-Assist people with disabilities to increase their income without increasing their dependency on income transfers -More accessible services
Lombe et al. 2016 (US) [72]	To explore the saving behavior, barriers and facilitators of participating in Personal Options program, a consumer directed care program for people with disabilities	29 people with disabilities (mean age 63, 79% female) -income and disability type not reported	Survey (saving behavior, barriers and facilitators, program effects)	-Participants were able to save money through the program to enhance their welfare and quality of life -79% of participants felt their resource consultants were helpful -96% reported positive program effects	-Restrictions in allowable goods and services is a barrier -Involve people with disabilities in the development of programs and policies -Consider the role that resource consultants play in connecting people with disabilities to programs
Mitra 2010 (South Africa) [63]	To assess South Africa’s disability cash transfer program	58,327 total survey respondents -2881 people with disabilities who received disability grant (mean age: 48.4; 48.6% male -55,446 people who did not receive the disability grant (mean age: 41.9) -income and disability type not reported	Survey (2005 General Household Survey) (economic well-being) 2001 Labor Force survey (labor participation, employment)	-Households receiving the disability grant are poorer than households not receiving the disability grant, and are worse off in the majority of indicators of economic wellbeing -The disability grant is going to households with more children and higher unemployment rates -Increased leniency in disability screening policy does not appear to have altered labor market behaviors	-Awareness campaigns about the disability grant and reduction of cost to apply for the disability grant in isolated areas would improve the exclusion error rate.
Mohapi 2016 (South Africa) [64]	To evaluate the sustainability of the social sector of expanded public works program as a poverty alleviation strategy	Survey: 152 officials -36 women and youth without disabilities -no sample characteristics reported	Questionnaire and focus group (access to education, health care)	-The social sector of the expanded public works programme has contributed to poverty alleviation by enabling participants to access education, health care, literacy -Participants also helped poverty reduction at a community level -87.4% of respondents agreed programme enabled access to education -72% agreed programme enabled access to healthcare and reduced illiteracy	-Program relies on volunteers and may not be sustainable -Partner with private sector when planning and implementing -Reach all areas of Gauteng province, especially for information dissemination -Greater training for officials and beneficiaries on sustainable development projects -Recruit more persons with disabilities and offer alternative work opportunities
Muhammad et al. 2022 (Nigeria) [60]	To examine Zakat (financial instrument) as a tool for alleviating poverty in Nigeria	10 participants (people with disabilities, parents of people with disabilities, and stakeholders) -no sample characteristics reported	Interviews (perspectives of financial instrument)	-Use of zakat income is a relevant tool for strengthening the skills and funding business ventures of vulnerable people (disabled) -People with disabilities sometimes do not receive Zakat and are missed due to lack of opportunities for community participation	-Expand Zakat by including consumable properties, cash, textiles, and entrepreneurial packages
Ngwakwe and Iqbal 2021 (South Africa) [65]	To examine the effect of social financial grant increases on poverty alleviation in South Africa	-no sample characteristics reported	Secondary analysis of social grant data (South African Social Security Agency)	-Only 3/7 types of grants (including disability grant) enhanced reduction in inequality -4/7 grants reduced poverty level (veterans, aid grants, grants for dependency, grants for fostering a child)	-Specific social grants can be used to target poverty and inequality reduction
Nuwagaba et al. 2012 (Uganda) [69]	To understand how people with disabilities access microfinance services in Bushenyi District of Uganda	39 participants (23 people with physical and sensory disabilities; 46% male) -mean age, income not reported	Interviews, focus groups, document review (perceptions of accessing services)	-People with disabilities can access microfinance services if they meet the requirements (savings, collateral, trustworthiness) -Access to microfinance services by people with disabilities is low (43%)	-Government policies and regulations should give special consideration to the circumstances of people with disabilities -Suggestions for improving access to microfinance for people with disabilities: engagement with community, self-initiative, enhanced education, training/inclusion for employees in finance
Opoku et al. 2019 (Ghana) [54]	To explore the impact of the Disability Fund on the lives of people with disabilities in Ghana	48 people with disabilities (58.3% women; mean age: 39) -income not reported	Interviews (perceived impact of disability fund)	-Many encountered barriers in accessing the fund: lack of information, delays in disbursement, insufficient funds -Only 7/48 reported benefiting from the fund, which helped support their education, businesses, acquire assistive devices	-Government should have better management and disbursement of funds
Prenovitz 2021 (US) [73]	To estimate the effect on initial wait time for social security on health, health care access and financial well being	2155 participants with various disabilities (mean age: 45.9; 47.6% male; 28.5% below federal poverty level) -income not reported	Secondary analysis of National Health Interview Survey data (wait time, financial well-being)	-Longer wait time decreases the likelihood that an applicant has benefits terminated and increases the likelihood that they are currently receiving benefits -Increased wait time associated with a greater number of conditions causing activity limitations -Wait time impacts poverty	-Focus beyond impact of wait time on employment and earnings to understand value and cost of program
Rupp et al. 2008 (US) [74]	To explore the role of supplemental security income in protecting against financial consequences of severe disability	-21,331 disability insurance only: (mean age: 41.6; 46.4% women, 3.4% poor) -5117 Supplemental Security Income only: (mean age: 32; 61.8% women) -8953 Serial Supplemental Security Income to Disability Insurance: (mean age: 34.2; 44.5% women, 16.7% poor) -1089 Joint Supplemental Security Income and Disability Insurance: (mean age: 29.3; 55.9% women, 39.5% poor) -4586 Not eligible for either disability insurance or SSI (mean age: 43.1 70.3% women, 7.7% poor)	Secondary analysis of survey of income and program participants (eligibility and insured status)	-Supplemental security income enhances the bundle of cash benefits available to people with disabilities -Interactions with other programs can enhance the safety net, especially in health insurance coverage	n/a
Sanson et al. 2018 (Guatemala, Nicaragua, Mexico) [55]	To describe the graduation program for people with disabilities in extreme poverty	936 households with people with disabilities -no sample characteristics reported	Mixed methods including participatory assessment and case studies, focus group (income, experience in program, savings, empowerment)	-positive outcomes in income generation and diversification, savings and food security -Improvements in empowerment and community participation, educational outcomes -Increased household income from 7% to 33% -People with disabilities lower graduation rates compared to people without disabilities	-Provide additional support to people with disabilities to achieve graduation criteria -Involve all household members in livelihood planning -Staff require guidelines and sensitivity training to make decisions about individual circumstances
Silverstein et al. 2024 (Malawi and Zambia) [25]	To understand whether cash transfer programs have different effects on children based on household disability status	Zambia data: -8052 children with and without disabilities -51% female -mean age: 6.36 -89% had some unmet material needs Malawi data: -9685 children with and without disabilities -49% female -mean age: 9.15 -88% had some unmet material needs unmet	Secondary analysis of evaluation data from 2 cash transfer programs (material well-being, nutritional status)	-Cash transfers improved access to material needs for all children -Reduction of illness in households with functional difficulties through cash transfers -Cash transfers improved nutritional status of children in households with disabilities	-More inclusion of households with disabilities in social protection programs
Trafford and Swartz 2023 (South Africa) [66]	To explore the perspectives from implementation officials of the care dependency grant for children with disabilities	5 grant implementation officials -no sample characteristics reported	Interviews (perspectives on care dependency grant)	-Some concerns about misuse of grants; confusion about the purpose of the grant -Difficulties related to permanence of the grant -Officials believe grant is used to cover care expenses and household costs -Assessments for grant qualification vary and formal diagnosis does not guarantee receiving the grant	-Greater distinction between grant recipients with temporary and permanent care needs -Offer shorter term social assistance to ease transition from child disability grant to adult disability grant and limit inclusion errors
Tschanz and Staub 2017 (Various European countries) [77]	To examine different disability policy models in European welfare regime	27 countries in the Eurobarometer -no sample characteristics provided	Secondary data analysis of OECD data (civil rights index: including anti-discrimination and accessibility; social protection and labor market integration)	-An activating and rehabilitation disability-model of disability policy is predominant in Nordic countries -Central European models indicates a preference for social protecting rather than activation and rehabilitation includes countries that normally have diverse welfare traditions	n/a
Vinck et al. 2019 (Belgium) [45]	To examine the non-take up of supplemental child benefits for children with a disability in Belgium	25,717 children with various disabilities, below age 21 years (67% boys) -13 parents (92% women) -25,057 without disabilities below age 21 years (51% boys) -income, mean age not reported	Secondary analysis of administrative database (non-use of benefits); and interviews (reasons for non-take up of benefits)	-Non-take up rate of benefits is at least 10%, mainly concerns children with less visible disabilities, resulting from insufficient information about the benefit and eligibility criteria, process costs -Requirements focus more on medical perspective, difficult for children with less visible disabilities	-More effort to providing frontline organizations and staff with information about benefits and eligibility criteria -More consideration of less apparent disabilities -More coherence in a disability policy package and improved communication
Vinck et al. 2022 (Belgium) [46]	To explore the connection between parental employment, social background and targeted cash support	17,677 children with disabilities ages 0–18 (mean age: 9.8) 16,206 children without disabilities ages 0–18 (mean age: 8.4) -gender, disability type and household income not reported	Secondary analysis of Datawarehouse labor market and social protection (poverty risk)	-Children with disabilities have a lower income poverty risk than non-disabled children -Each cash supplement has a poverty reducing impact for the children who receive them -Refundable tax credit reduces poverty risk more for disabled than for non-disabled	-More work on the non-take up of benefits
Vinck 2024 (Various European Countries) [76]	To explore the link between disability and poverty in a cross-national comparative analysis	4845 children with disabilities, aged 0–15 (mean age 8.9) (55.8% male) -94,437 without disabilities; 51.6% male) -disability type, household income not reported	Secondary analysis of European comparative data (child disability, child poverty)	-Many differences across Europe in the poverty-reducing effectiveness of social transfers -Social transfers achieve more for children with disabilities in more than half of European countries	n/a
Wang et al. 2023 (China) [49]	To explore levels of multidimensional poverty of people with disabilities and poverty reduction effect of employment services	435,312 persons with multiple disabilities aged 16–59 years -no other sample characteristics reported	Secondary analysis (multidimensional poverty: economy, health, education, insurance, social participation)	-About 90% of people with disabilities are deprived of at least one dimensions and 30% are in a state of severe multidimensional poverty -Employment services have a significant improvement effect on multidimensional poverty	n/a
Weathers and Hemmeter 2011 (US) [75]	To explore the impact of changing financial work incentives on earnings of social security disability insurance beneficiaries	1820 total participants -923 treatment group (mean age: 39.7; 51.9% male) 897 control group (mean age: 39.62; 49.2% male) with various disabilities -income not reported	Random assignment into benefit group or control group (earnings, social security disability insurance benefits)	-The new pilot program led to a 25% increase in the percentage of beneficiaries with earnings above the annualized substantial gainful activity amount; it did not result in a reduction in benefit payments	n/a
Wootton et al. 2024 (South Africa) [67]	To describe protective effect of disability benefits against lost income for South Africans with Schizophrenia	1154 individuals with schizophrenia (13.7% female, mean age: 36.3; 94% unemployed) -income not reported	Case control study (household wealth, financial hardship, poverty)	-Receipt of disability benefits are significantly associated with increased household and personal wealth	-Harmful to encourage employment by withholding disability benefits from people living with chronic schizophrenia
Yan et al. 2023 (China) [50]	To assess the effects of targeted poverty alleviation policy on poverty-stricken households	796 households 60 poverty-stricken due to disability -no sample characteristics reported	Questionnaire (income, disability, labor, fund, education)	-Houses with a member with a disability had the lowest income among the 6 groups	-To ensure the stability of poor households, relevant associated aid policies need to place different emphases based on household characteristics

* Note that we only report on sample characteristics and findings related to our research question. We also use the terminology from the original articles and recognize that there have been changes over time and across cultures.

### Overview of themes

Types of poverty alleviation strategies included amongst the studies in this review were: (1) poverty alleviation policies; (2) programs and practices to address poverty; and (3) cash transfers.

#### Theme 1: Poverty alleviation policies.

Five studies described various poverty alleviation policies in Brazil, China, Italy, Malawi and various European countries (i.e., disability pension policy [57], Disabilities Act [48], poverty alleviation policy [50], national strategy for financial inclusion [59], and disability policy [77]). For example, Tschanz and Staub [77] examined various disability policy models in European welfare regimes and discovered four distinct disability policy models. They highlighted that an activating and rehabilitation disability model of disability policy is predominant in Nordic countries [77]. They discovered that central European disability policy models preferred social protection rather than activation and rehabilitation, including countries that normally have diverse welfare transitions [77]. Further, Agovino and Ferrara [57] assessed whether civilian disability pensions in Italy could address poverty. In the long-term, they observed that a minimum increase of civil disability pensions would reduce income inequality and allow

households with a disabled member to consume more and exit from poverty [57]. As well, Lamont et al. [48] explored how the Disability Act in Brazil affected self-employment income and those with disabilities who are poor. Income levels of employed people improved after the Act, with the income gap between employed and self-employed individuals with disabilities growing wider after the law was passed [48]. The law mostly benefited poor people with disabilities who were unable to find employment [48]. Meanwhile, Jiya et al. [59] explored the preparedness and accessibility of financial services for people with disabilities in a small sample in Malawi after the implementation of a national strategy for financial inclusion, specifically targeting those participating in financial services. They explained that barriers to financial inclusion still exist within the business model of banks due to inaccessible technological resources and communication barriers [59].

Regarding policy challenges, several studies discussed the lack of representation, inclusion, and accessibility for people with disabilities in policy creation and implementation [45,51,53,57,59,64,69,72]. For instance, Nuwagaba et al. [69] found that limited consideration given to people with disabilities in government policies in Uganda. Studies in our review pointed to the need for more alternative strategies and policies to addressing poverty for people with disabilities [45,50,57,59,61,69,72]. Others noted that political structures should be separate from the management and disbursement of policies to avoid potential conflicts [53]. Two studies underscored the importance of adapting policies to meet the specific needs of people with disabilities [51,69]. Policies created to address poverty alleviation require more coherence and clearer communication [45]. Another study highlighted how essential it is to involve people with disabilities in the development of programs and policies [72]. Further, the longer-term sustainability of programs and policies should be explored [64].

#### Theme 2: Poverty alleviation programs and practices.

Twelve studies focused on various types of poverty alleviation programs and practices including: microfinance programs [51,69], financial service practices [59], social entrepreneurs [68], the consumer directed care program [72], employment services and vocational programs [49,70], work incentives [61,75], the social sector of expanded public works program [64], housing interventions [71], and the graduation program [55].

**Benefits of poverty alleviation programs:** Seven studies described the beneficial impacts of poverty alleviation programs including improvements in multidimensional poverty [49], educational opportunities and outcomes [49,55,64,68], employment [61], increased income [55,75], savings [55,72], food security [55,64], social/community participation [49,55], quality of life [72], independence [55], and self-esteem [55]. For example, Wang et al. [49] explored levels of multidimensional poverty of people with disabilities and the poverty reduction effect of employment services in China. They found that employment services had a significant improvement on multidimensional poverty, which was reflected in education, insurance, and social participation [49]. Moreover, Kostol and Mogstad [61] explored how financial incentives induce disability insurance recipients to return to work among a Norwegian sample. They described that financial work incentives can encourage people with disabilities to gain employment [61]. Additionally, Weathers and Hemmeter [75] explored the impact of changing financial work incentives on earnings of social security disability insurance beneficiaries in a US sample. The new pilot program led to a 25% increase in the percentage of beneficiaries with earnings above the annualized substantial gainful activity amount [75].

In Sanson et al.’s [55] exploration of the graduation program for people with disabilities in extreme poverty in Guatemala, Nicaragua, and Mexico, they reported positive outcomes in income generation, diversification savings, and food security. Improvements in independence, self-esteem, community participation, and educational outcomes were also noted [55]. Meanwhile, Mohapi [64] evaluated the sustainability of the social sector of expanded public works program as a poverty alleviation strategy on a small South African sample. They found that the program contributed to poverty alleviation by enabling participants to access education, literacy, and health care [64]. They also explained that the program helped to reduce poverty at community (e.g., food gardens) and individual levels (e.g., buying in bulk) [64].

Lombe et al. [72] explored the saving behaviours of those participating in the “Personal Options Program”, a consumer directed care program for people with disabilities in a small US sample. Participants saved money through the program to enhance their welfare and quality of life [72]. Within this program, 79% of participants reported that resource consultants helped with decisions to save money in the program and 96% described positive program effects [72]. Additionally, Connor and Bent-Goodley [68] highlighted how social entrepreneurs acted as a poverty alleviation strategy in Tanzania. Their study noted that women social entrepreneurs were successful in building educational opportunities for girls and low-income women. They evolved in business to address change and economic growth while empowering vulnerable populations and people with disabilities [68].

**Barriers and factors affecting access to poverty alleviation programs:** Three studies described barriers or factors affecting access to poverty alleviation programs for people with disabilities. Common barriers included limited access [69], inaccessible financial services [59], communication barriers [59], and sociodemographic characteristics [51]. For example, Nuwagaba et al. [69] explored how people with disabilities access microfinance services in Bushenyi District, Uganda and found overall poor access.

Socio-demographic characteristics also affected access to poverty alleviation programs. For instance, Beisland and Mersland [51] explored characteristics of disabled versus non-disabled clients in a microbank program in Ecuador. They discovered that disabled clients were more often male, single, had fewer children and were older compared to those without disabilities [51].

#### Theme 3: Cash/income transfers for poverty alleviation.

Twenty-two studies focused on cash or income transfers as a mechanism for poverty alleviation, which presented in the following forms: social transfers [23], social security [71,73], cash benefit program [47], disability allowance [24,58], disability fund [52–54], cash transfer [22,25,46,63,66,67,76], disability grants [62], income transfers [56], Supplemental Security Income (SSI) [70,74], financial grants [65], and supplemental child benefits [45]. These studies focused on the impact and/or factors affecting receipt of cash transfers.

**Impact of cash/ income transfers:** Seventeen studies described findings related to the impact of cash transfers in Belgium [46,76], Ghana [52–54], Kenya [22], Korea [56], Malawi [25], the Maldives [58], South Africa [62,63,65,67], the US [70,71,74], Zambia [25] and other various countries [23]. The majority of the studies (13/17) observed a positive impact in reducing poverty [22,23,46,56,58,65,67,71,74,76], inequality [65], while enhancing material needs including assistive devices [54], food security [25,54,58], access to health care [22] and other safety nets [62,71,74], supports [62], health insurance coverage [74], education [54], work/productive activities [22,54], wellbeing [58], independence [62], and empowerment [22]. For example, Caminada et al. [23] highlighted that 15% of the population is helped out of poverty through income tax benefits. For the working-age population, disability/survivor benefit transfers contribute the most poverty reduction, at 81% [23]. Additionally, Rupp et al. [74] found that eligibility for Supplemental Security Income enhanced the bundle of cash benefits available to people with disabilities in the US through its interaction with other programs such as Disability Insurance and Medicaid. They also noted that interactions with other programs can enhance the safety net, especially in health insurance coverage [74]. Among households with a disabled person in a Korean sample, Lee and Choi [56] reported that receiving income transfers decreased the poverty rate by 55.9% and the poverty gap by 84.8%. Their findings suggest that means-tested public income transfers were more effective in reducing poverty levels than social insurance or private income transfers [56]. Additionally, the provision of means-tested programs was more likely to decrease the probability of experiencing poverty than social insurance and private income transfers [56].

Of the studies that discussed how cash transfers were used, they described spending it on assistive devices and disability aids [53,54], education [52–54], and productive activities or investments [22,52,54]. Several studies highlighted how cash transfers were used to help address daily needs and as extra household income [22,25,53,56,62,71]. For example, Kelly [62] explored the influence of disability-related grants on family practices and household composition in a small South African sample and found that disability grant income is often shared within households. The contribution of a stable income can provide opportunities for people with disabilities to act with agency and to secure care and support [62]. Silverstein et al. [25] reported that cash transfers improved access to material needs for all children in Malawi and Zambia. Similar trends were noted in Abdille and Mbataru [22]’s study who found that cash transfers supported income, health care access, investment in productive activities and empowerment, which had a significant economic impact on the well-being of people with disabilities. Wootton et al. [67] explored the protective effect of disability benefits against lost income for South Africans with disabilities and reported that receiving disability benefits was significantly associated with increased household and personal wealth.

In a cross-national comparative analysis, Vinck et al. [76] found many differences across Europe in the poverty reducing effectiveness of social transfers, which achieved more for children with disabilities in more than half of European countries. When controlling for parental employment and social background, the income poverty risk did not differ significantly between children with and without disabilities [46]. Vinck et al. [46] found that each cash supplement reduced the poverty risk for children (with and without disabilities) who receive them. Additionally, when they examined the impact of the supplemental child benefit and refundable tax credits, the income poverty risk among children with disabilities was significantly lower than among children without disabilities [46]. The authors contend that this could be a result of those who receive the supplemental benefit being more likely to live in poverty due to their lower parental employment and disadvantaged social background [46]. Further, families with disabled children, on average, get higher regular child benefits than families without disabled children and their tax credit is higher [46].

Some other, non-income related benefits of cash transfers were highlighted in various studies. Research on the Disability Fund in Ghana showed that the fund had positive outcomes on the livelihoods of people with disabilities including inclusion into the community, enhanced dignity and feeling valued [52], which assisted them with purchasing essential items [53], and helped to support their education, businesses, and to acquire assistive devices [54]. Social financial grants (including the Disability Grant) in a South African sample enhanced reduction in inequality [65]. Meanwhile, Banks et al. [58] explored the impact of disability allowance on financial wellbeing in the Maldives and found a modest impact of disability allowance on financial well-being, mostly in food security. Some found that cash transfers have the potential to improve household resources and food security, alleviate poverty and reduce household financial burden [22,25].

Two studies reported no impact of cash transfers [58,63]. For example, Mitra [63] discovered that increased leniency in disability screening policy, in a South African cash transfer program, did not alter market behaviours. Further, Banks et al. [58] found that the disability allowance did not reduce the poverty gap [58]. Finally, two studies observed a negative impact of receiving a disability grant in South Africa, which involved family conflict, pressure to retain the grant and potential for neglect and abuse [62]. Additionally, Trafford and Swartz [66] explored the perspectives of implementation officials of the care dependency grant for children with disabilities in South Africa and they reported some concerns about the misuse of grants. Limits to the legislation also existed around the grant and confusion about the purpose of the grant [66].

**Factors affecting cash transfers:** Twelve studies described factors affecting cash transfers for people with disabilities in Belgium [45], Brazil [47], Ghana [52–54], the Maldives [24], South Africa [63,66,67], and the US [70,71,73]. Aspects affecting cash transfers included lack of information [24,45,53,54,66], barriers in eligibility criteria [24,63], wait time for social security benefits [73], delays in disbursement [53,54], insufficient funds [52–54], perceptions of disability [24], disability type [24,45], income [24,67], education [47,67], geographic location [24,67], gender [24,47] and age [24,47]. For example, a higher number of household assets, higher level of education, current employment, higher number of household members and living in an urban area was significantly associated with receipt of a disability grant in a South African sample [67]. Factors affecting the uptake of the disability allowance included lack of information, stigma/perceptions of disability, eligibility criteria, geographical and financial factors [24]. Vinck et al. [45] found that the non-uptake rate of supplemental child benefits for children with disabilities in Belgium was at least 10%. Concerns existed for children with less apparent disabilities, resulting from insufficient information about the benefit and eligibility criteria and process costs [45].

Regarding the Disability Fund in Ghana, Karimu et al. [52] noted some barriers of the District Assemblies Common Fund program, including the quality of purchased items, procurement issues, disparity in allocations, and lack of transportation support. Other challenges with the Disability Fund included lack of information, difficulty accessing the fund, insufficient amount of funding, and disbursement delays [53,54].

Socio-demographic characteristics also affected cash transfers. For example, Hameed et al. [24] observed that disability allowance coverage in the Maldives was lowest for women, older adults, people living in the capital city, wealthier households and people with sensory impairments [24]. Further, Campos et al. [47] examined who benefits from a continuous cash benefit program for children and youth with disabilities in a Brazilian sample and found a predominance of male, preschoolers and illiterate individuals in the program.

## Discussion

Our review explored policies, practices, and interventions that address poverty among people with disabilities. Focusing on poverty among people with disabilities is critical because it can impact health, social, and educational outcomes [78]. To enable a more inclusive society, further targeted supports are required to reduce poverty [27,54]. Our review highlighted the breadth of interventions, policies, and programs aimed at poverty alleviation for people with disabilities globally, demonstrating mixed results.

Our findings emphasize the potential impact of some poverty alleviation policies, programs, practices, and cash transfers for people with disabilities. For example, some research shows a link between individual income and improved educational, financial, and health outcomes [20,55,79]. Other research highlights that cash transfers aim to increase income and reducing poverty, help to relieve financial pressure while improving food security [58], socio-economic independence and health outcomes [20,53]. Further research is required to better understand the longer-term impacts of poverty reduction strategies, the components of effective interventions, how cash transfers are being used, and who is most likely to benefit.

Our review also noted factors affecting access and receipt of poverty reduction strategies. There are many socio-structural barriers for people with disabilities to access poverty alleviation resources, including lack of information and awareness about poverty reduction resources, difficulties accessing the funds, disbursement delays, and inadequate funding. As well, some studies expressed concerns about eligibility criteria, expensive and inaccessible medical exams required for eligibility, and fear of receiving disability-related stigma from their community [24,59]. Restrictions in eligibility and in what funds can be used for, limits the effectiveness of the policies and practices [45,72]. Lacking access to poverty reduction strategies can have longer-term repercussions throughout the life course [9]. Although some programs and policies may help to tackle poverty, they might lack the proper management and communication to optimize effectiveness for people with disabilities [24,52]. Our review demonstrates a need for improving awareness of the various anti-poverty initiatives and resources for people with disabilities and the staff supporting them.

Poverty alleviation policies, practices, and programs can also support people with disabilities who encounter food insecurity [53,55,58,64]. Doing so is critical, as the World Health Organization [13] notes that a greater number of households with a disabled person often lack access to food compared to households without a member with disabilities. The United Nations Sustainable Development Goals include ending food insecurity, indicating this issue is of key global importance and requires immediate action [5]. Initiatives such as disability funds or grants [53,58] and anti-poverty programs [55] could help people with disabilities to afford the food and nutrition they need to maintain their health and wellbeing. Anti-poverty initiatives could also lead to an increase in income, which could enhance food security [55].

One of the primary outcomes of poverty alleviation strategies used for people with disabilities involved ensuring their economic well-being. Such strategies often come in the form of increased income, addressing income gaps, teaching financial responsibility and financial literacy, and saving behaviours. Most of the studies in our review found that the policies, programs, and interventions had beneficial impacts towards the economic wellbeing of people with disabilities. However, we also noted that people with disabilities often experience barriers to accessing financial programs and institutions due to discrimination and perceived risk [51,59,69]. This in turn, limits their ability to exit poverty and achieve financial inclusion in their communities [59].

Receiving support through poverty alleviation policies, practices, and programs can lead to a greater sense of empowerment, social inclusion, and overall well-being among people with disabilities [10,22,52,55,68]. Cash transfers had a positive impact on the economic wellbeing of people with disabilities in Kenya, as they provided individuals with funds to invest in productive activities and feel empowered [22]. Our review featured poverty alleviation endeavours in Ghana and Brazil that focused on ensuring people with disabilities can invest in business ventures or to maintain self-employment. Entrepreneurship and small businesses were a tool for people with disabilities to provide for themselves in a way that promotes autonomy and economic empowerment [48,52,54]. Individuals with disabilities enrolled in Ghana’s Disability Fund also felt valued, greater inclusion into the community, and enhanced dignity [52]. As researchers demonstrate, anti-poverty initiatives are essential to building social inclusion, and greater supports are needed for people with disabilities [14,22].

An important aspect that was notably absent in the studies within this review was the impact of belonging to multiple marginalized intersectional identities (e.g., racialized, gender minoritized). People who belong to multiple minoritized groups often experience additional challenges or barriers in accessing poverty reduction strategies; however, further research is needed. It is also important to consider how opportunities to obtain educational credentials and employment vary greatly by country (especially high-income versus lower-income countries). Many people with disabilities in lesser developed countries often experience incredible challenges in accessing education due to physical and attitudinal barriers. A lack of access to such opportunities could exacerbate poverty [80].

### Implications

The findings of our review underscore several important implications for policy, programs, and practices. Regarding policies, some of the studies in our review suggested the need for more alternative strategies and policies to addressing poverty for people with disabilities [57]. Others note that political structures should be separate from the management and disbursement of policies and programs to avoid conflicts [53]. Bonga [81] argues that a comprehensive approach for poverty alleviation is needed that is rooted in theories of poverty, empowerment, social inclusion, and bottom-up approaches by decision-makers to help address the challenges encountered by minoritized groups. Thus, it is important to involve people with disabilities in the development of programs and policies that can help to alleviate poverty [82].

Some studies pointed out the demand for greater funds dedicated to disability funds [52]. For example, some emphasize that governments should consider increasing the monthly disbursements given to people with disabilities [22]. Cash transfer programs could also consider partnering with other programs to extend the financial and related nonfinancial supports to people with disabilities [22]. Additionally, it would be worthwhile for cash transfer programs to include follow-up programs so that people with disabilities can receive support to enhance their income [22]. Further, restrictions in allowable goods and services covered through cash transfer programs could be reconsidered to positively affect the quality of life of people with disabilities [72].

Our findings highlighted that people with disabilities need enhanced financial literacy and financial management training [53,59], and improved access to financial services and microfinance [59,69]. Having accessible and inclusive financial services and financial literacy is essential for reducing poverty for people with disabilities [59]. This should be supplemented by enhanced education and training for employees in finance regarding inclusion and the needs of people with disabilities [69].

Our review suggested that more effective communication could facilitate poverty reduction and the uptake of poverty alleviation programs. Enhanced communication about the availability of policies, programs, and services to people with disabilities is necessary. Further efforts are required to provide frontline organizations and staff with information about benefits and eligibility criteria of relevant programs [45]. Some researchers argue that raising awareness about the existence of social protection programs and how to apply is important, but likely insufficient for improving uptake and coverage without additional efforts to widen the knowledge of eligibility criteria and addressing stigma of receiving such benefits [24]. Navigating different policies, programs, and practices can be overwhelming for people with disabilities, especially when the inclusion criteria is unclear or restricted, and a program has multiple barriers to entry [24,45,72]. It is important to consider the role of resource consultants as an intermediary to support people with disabilities, and as a means to spreading awareness and information regarding available programs and services [72]. All those involved in developing or delivering anti-poverty interventions should undergo training to learn about the intersection of disability and poverty, to better understand the needs of this population [9]. Overall, increased communication between decision-makers, people implementing the programs, and people with disabilities is needed to develop and implement effective poverty alleviation strategies that empower and include the voices of those using the program [81,82]. Doing so could also help to ensure that clearer, targeted, and sustained supports exist for people with disabilities who are disproportionately impacted by poverty [45,50]. It is imperative to adapt programs and policies to meet the specific needs of people with disabilities (while considering how their needs vary by age and disability type) [51,69] and to involve them in the development of programs and policies [72]. Engagement with local communities is critical to addressing and promoting economic stability for people with disabilities [68].

Our findings underscore the urgent need for more accessible services, education, and employment opportunities to help increase income and in turn, reduce poverty [52,56]. However, it is vital that we assist people with disabilities to increase their incomes without increasing their dependency on income transfers [56]. Some researchers contend that despite the importance of cash transfers for people with disabilities they are likely insufficient on their own to enhance financial security [58]. Additional or more tailored policies, programs and interventions may be needed to address the inequalities experienced by people with disabilities such as addressing stigma and discrimination, inaccessible environments, and the availability and affordability of services [58]. Further targeted, sustained support for people with disabilities is required to help with poverty alleviation [50].

### Limitations and future research

There are several limitations to consider in this review. Many of the studies lacked details on participant characteristics, which makes it difficult to understand who is, and is not, accessing and benefiting from poverty alleviation strategies. Our review includes a broad range of disabilities and ages, and further research is needed to understand whether specific policies and programs work better for certain age groups (child-onset versus adult) and types of disabilities (including preventable versus non-preventable; apparent versus no-apparent [83]). Further studies could also take a more in-depth analysis of specific types of disabilities and how this intersects with poverty. Given that this review spanned across many countries (both high and low-income), we also recognize the cultural variations in experiences of poverty and treatment of people with disabilities, and the related supports and resources (i.e., poverty alleviation policies) available to them. We acknowledge that definitions of disability and poverty vary widely across the studies included in our review where some studies used census bureaus and federal poverty lines to define poverty while others used the OECD scale or the World Bank Standard. Therefore, caution should be used when interpreting the findings. Future studies should consider incorporating multidimensional poverty frameworks and explore how non-monetary dimensions, such as education, health and living standards can also affect poverty. Additional comparative analysis of poverty thresholds used across various countries (both high and low-income) are also needed. Further research could also consider exploring the impact of cash transfers on expenditures, and specifically how they benefit people with disabilities and their households.

Despite not having a date restriction for our search, our review only found articles from the past 16 years. Additionally, there were many studies involving descriptions of poverty alleviation policies and programs that were excluded from our review because they were not empirical. We encourage researchers to evaluate the efficacy of poverty alleviation strategies so we can better understand what might work best to support people with disabilities. Regarding future research, more evaluation of the effectiveness of policies geared towards poverty alleviation of people with disabilities are needed. Researchers should explore what the essential key components of programs and policies are; how they work, and for whom (e.g., age, type of disability, geographic location). As well, more consideration of intersectionality and especially people with multiple minoritized identities (e.g., disabled, low socio-economic status, racial minoritized.) is needed in addition to their lived experiences of poverty and financial strain [80]

## Conclusions

Our review explored policies, practices, and interventions to address poverty among people with disabilities. The studies within our review highlighted the benefits, barriers and factors affecting poverty alleviation policies, programs, and practices. Cash transfers are a common form of poverty reduction strategy for people with disabilities. Anti-poverty initiatives have the potential to contribute to improved educational and employment opportunities, healthcare access, income, food security, psycho-social well-being, and quality of life among people with disabilities. Although many of the poverty reduction strategies that we reviewed showed potential, there is an urgent need for the further development of policies and programs to address the socio-economic needs of people with disabilities.

## Supporting information

S1 File Sample search strategy. (DOCX)

S2 File PRISMA-ScR Checklist. (DOCX)
