# Supplementary material for: Poverty alleviation policies, programs and practices for people with disabilities: A scoping review and recommendations
Source: PLoS One. 2025 May 13;20(5):e0323540. doi: 10.1371/journal.pone.0323540 (PMC12074602; doi:10.1371/journal.pone.0323540)
Supplement: S1 File — (DOCX) [file pone.0323540.s001.docx]

**S1. Sample search strategy**

**Database: Ovid MEDLINE(R) ALL <1946 to June 21, 2024>**

**1**  disability.mp. (295440)
**2**  disabled.mp. or exp Disabled Persons/ (105365)
**3**  functional limitation.mp. (2911)
**4**  physical impairment.mp. (2005)
**5**  handicap.mp. (13241)
**6**  sensory impairment.mp. (2138)
**7**  motor disorder.mp. or exp Motor Disorders/ (2401)
**8**  wheelchair user.mp. (269)
**9**  cerebral palsy.mp. or exp Cerebral Palsy/ (33421)
**10**  spinal cord injury.mp. or exp Spinal Cord Injuries/ (71300)
**11**  congenital disorder.mp. or exp "Congenital, Hereditary, and Neonatal Diseases and Abnormalities"/ (1400296)
**12**  amputation.mp. or exp Amputation, Surgical/ (56916)
**13**  exp Hydrocephalus/ or hydrocephalus.mp. (39879)
**14**  scoliosis.mp. or exp Scoliosis/ (30977)
**15**  developmental disability.mp. or exp Developmental Disabilities/ (24003)
**16**  exp Autism Spectrum Disorder/ or exp Autistic Disorder/ or neurodevelopment disorder.mp. or exp Intellectual Disability/ or exp Neurodevelopmental Disorders/ (261393)
**17**  brain injury.mp. or exp Brain Injuries/ (124663)
**18**  orthopaedic condition.mp. (83)
**19**  1 or 2 or 3 or 4 or 5 or 6 or 7 or 8 or 9 or 10 or 11 or 12 or 13 or 14 or 15 or 16 or 17 or 18 (2167320)
**20**  welfare.mp. (94197)
**21**  social security.mp. or exp Social Security/ (15650)
**22**  income transfer.mp. (73)
**23**  exp Food Assistance/ or supplemental income.mp. (2021)
**24**  cash transfer.mp. (853)
**25**  poverty reduction.mp. (854)
**26**  social grant.mp. (23)
**27**  income assistance.mp. (132)
**28**  social assistance.mp. (1056)
**29**  poverty intervention.mp. (9)
**30**  20 or 21 or 22 or 23 or 24 or 25 or 26 or 27 or 28 or 29 (113065)
**31**  19 and 30 (9443)
